# Chromosomal Variations and Clinical Features of Acute Lymphoblastic Leukemia in the North Indian Population: A Cross-Sectional Study

**DOI:** 10.7759/cureus.60451

**Published:** 2024-05-16

**Authors:** Anam Ahmad, Alka Dwivedi, Sushma Tomar, Akriti Anand, Rakesh K Verma, Archana Rani, Rakesh K Diwan

**Affiliations:** 1 Anatomy, King George's Medical University, Lucknow, IND; 2 Clinical Hematology, King George's Medical University, Lucknow, IND

**Keywords:** translocation, philadelphia-positive, hemoglobin, b-cell acute lymphoblastic leukemia, acute lymphoblastic leukemia

## Abstract

Background: The key prognostic markers in acute lymphoblastic leukemia (ALL) include age, leukocyte count upon diagnosis, immunophenotype, and chromosomal abnormalities. Furthermore, there was a correlation between cytogenetic anomalies and specific immunologic phenotypes of ALL, which in turn had varied outcomes. The objective of this study was to examine the occurrence of cytogenetic abnormalities in individuals diagnosed with acute lymphoblastic leukemia.

Methods: The study employed a cross-sectional design to investigate genetic evaluation and clinical features in 147 ALL patients between March 2021 and August 2022. Demographic data (like age and sex), clinical manifestations, and hematological parameters were collected. Cytogenetic analysis (G-banding) was performed to identify chromosomal abnormalities. The mean±SD and analysis of variance (ANOVA) were used to assess associations and differences among variables using SPSS Version 24 (IBM Corp., Armonk, NY, USA).

Results: The study shows male n=85 and female n=62 in ALL patients, with prevalent clinical manifestations: fever n=100 (68.03%), pallor n=123 (83.67%), and lymphadenopathy n=65 (44.22%). The hematological parameters like hemoglobin (Hb) (6.14±2.5 g/dl), total leukocyte count (TLC) (1.7±1.05 cell/mm^3^), and platelet count (1.2±0.11 lac/mm^3^) show a significant variation (P<0.05) in patients aged 30-50 years. In addition, chromosomal abnormalities, particularly 46, XX, t(9;22), were prevalent, emphasizing the genetic heterogeneity of ALL.

Conclusion: The study shows a male predominance with ALL, prevalent clinical manifestations, and significant hematological parameter variations in the 30-50 age group. Chromosomal abnormalities, notably 46, XX, t(9;22), underscore the genetic complexity of the disease, which necessitates tailored therapeutic interventions informed by genetic profiles.

## Introduction

Acute lymphoblastic leukemia (ALL) is derived from lymphoid hematopoietic progenitors. The condition arises from a single progenitor cell of either the T-lymphocyte or B-lymphocyte lineage [1]. The increase and extensive presence of blast cells in the bone marrow (BM) impairs hematopoiesis, causing anemia, thrombocytopenia, and neutropenia [2]. The correlation between age and gender has been firmly established in determining the incidence rate of ALL. Earlier studies indicated that the prevalence of ALL was highest among children aged one to four years, followed by a significant decrease, and reached its lowest point among adults aged 25 to 45 years. Approximately 60% of all instances are identified before reaching the age of 20 [3]. ALLs are malignant tumors made up of undifferentiated B (pre-B) or T (pre-T) cells called lymphoblasts [4]. Even though the disease is most common among children, accounting for up to 80% of all leukemias in that age group, it can strike anyone at any moment. ALL is classified into several subtypes using morphologic, immunologic, cytogenetic, and molecular genetic approaches [5], whereas B-lineage ALL (B-ALL) accounts for approximately 85 percent of pediatric acute “leukemias” [6].

A number of cytogenetic subgroups in juvenile acute ALL are routinely used to classify patients for specific treatment approaches [7]. Despite extensive medical treatment, a significant minority of people continue to experience a lack of recovery. Certain cancer patients may receive overly aggressive treatment, resulting in undue suffering from chemotherapy and radiation therapy [8]. The first karyotype is widely acknowledged as a highly reliable prognostic predictor, prompting the development of genotype-specific pharmacological therapies [9]. As a result, the purpose of this study is to look into the prevalence of chromosomal abnormalities in the North Indian population. The purpose of this study is to help us understand the genetic basis of clonal evolution and relapse, as well as the function of inherited genetic differences in leukemia formation. The results of this study have the potential to make significant contributions to the domains of oncology and hematology. They can improve the policy plan and epidemiological status of the ALL. These insights can be used as a helpful resource to guide the development of specific therapeutic approaches.

## Materials and methods

A cross-sectional study was conducted at the Cytogenetic Laboratory of the Department of Anatomy and Department of Clinical Hematology, King George’s Medical University, Lucknow, to evaluate the genetic and clinical features of ALL patients. The protocol was approved by the Institutional Ethics Committee and follows the Declaration of Helsinki (Reference Code 101 ECM II B Thesis/P35 Dated 1/6/2020), and a total of n=147 ALL patients within the predefined sampling frame were included, and n=18 patients were lost in follow-up of the study. Participants were recruited from the Department of Clinical Hematology, King George’s Medical University, between March 2021 and August 2022. Inclusion criteria encompassed patients diagnosed with ALL within a predefined age range (<30, 30-50, and >50 years), provided written consent, demographic details, and clinical data, including hematological parameters. The exclusion criteria involved patients diagnosed with other types of leukemia or hematological malignancies, outside the predefined age range, individuals with incomplete medical records, and patients unable to comply with study procedures or follow-up requirements, were also excluded from participation.

Data collection

The data were collected in a predesigned questionnaire (see Appendix). Demographic variables, clinical features, and hematological parameters were collected from medical records and patient interviews. Demographic variables included age, gender, and smoking. Clinical features, such as fever, pallor, lymphadenopathy, rashes, joint pain, headache, and bleeding manifestations, were documented based on patient reports and physical examinations. Hematological parameters, including hemoglobin (Hb), total leukocyte count (TLC), and platelet count, were measured using standard laboratory techniques. Cytogenetic analyses of blood were performed in institutional cytogenetics laboratories, and karyotypes were centrally reviewed biannually. Peripheral blood was obtained at diagnosis from all patients and processed using unstimulated 72-hour cultures, a direct method, or both. G-banding was usually done. The cytogenetic clone and karyotype were described according to the guidelines of the International System for Human Cytogenetic Nomenclature [10]. A minimum of 20 metaphase cells were examined in patients classified as having a normal karyotype [11,12].

Statistical analysis

Descriptive statistics were used to provide a concise summary of demographic characteristics, clinical aspects, and hematological parameters. Categorical variables were displayed as frequencies and percentages, while continuous variables were displayed as mean±standard deviation (SD). The chi-square test or Fisher's exact test was employed to examine categorical data, while the analysis of variance (ANOVA) or student's t-test was utilized to compare continuous variables between groups. The threshold for statistical significance was established at a p-value of less than 0.05 using SPSS Version 24 (IBM Corp., Armonk, NY, USA).

## Results

A total of n=147 ALL patients falling in our sampling frame were included in the study, and genetic evaluation was done. In a study, male n=85 (58%) and female n=62 (42%) were present in ALL patients. The demographic variables along with the clinical features of ALL patients are summarized in Table 1. In addition, the standard metaphases were not identified in the cytogenetics findings and were classified under the not obtained category.

**Table 1:** Characteristics of patients. N: numbers, %: percentage.

Variable name	Characteristics	Numbers	Percentage
		(n)	(%)
Numbers (n)		147	100
Sex	Male	85	58
	Female	62	42
Age (years)	Mean±SD	52.91±10.01	
	<30	62	42
	30-50	55	37.4
	>50	30	20.4
Smoking	Yes	84	57.14
	No	63	42.85
Chromosomal abnormalities	46,XX	21	14.29
	46,XY	28	19.05
	46,XY,del11(q23)	3	2.04
	46,XY,t(7;15)	2	1.36
	46,XY,t(9;22)	13	8.84
	47,XX+18	2	1.36
	48,XX,del(9)(p22),+12,+21	3	2.04
	52,XY,+1,+8,+10,+17,+21,+21,+22,+mar1	4	2.72
	45,XY,del(7)(p15),9,der(9),i(9)(q10),t(9;22)	8	5.44
	46,XX,t(9;22)	15	10.20
	47,XY,-7,+8,+21,t(9;22)	4	2.72
	46,XX,t(1;19)	4	2.72
	Not obtained	40	27.21

Complication status

**Figure 1:**
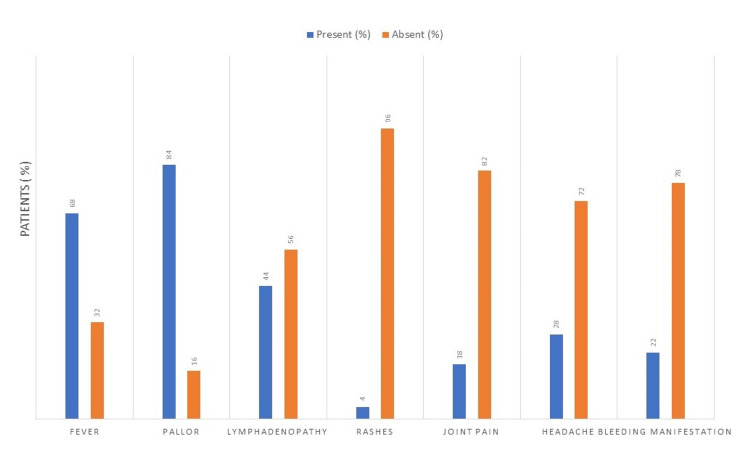
Presentation of complication status in the study population. %: Percentage.

In the study population, the complication status was presented as fever. Among the participants, 100 (68.03%) reported experiencing fever, while 47 (31.97%) reported no fever. Pallor: a total of 123 participants (83.67%) exhibited pallor, while 24 participants (16.33%) did not exhibit pallor. Lymphadenopathy: out of the participants, 65 (44.22%) had lymphadenopathy, while 82 (55.78%) did not. Rashes: Only six participants (4.08%) reported having rashes, whereas 141 participants (95.92%) did not. Joint pain: 27 participants (18.37%) experienced joint pain, while 120 participants (81.63%) did not. Headache: among the participants, 41 (27.89%) reported having headaches, while 106 (72.11%) did not. Bleeding manifestations: 32 participants (21.77%) reported bleeding manifestations, whereas 115 participants (78.23%) did not (Figure 1).

Hematological parameters and age groups

**Figure 2:**
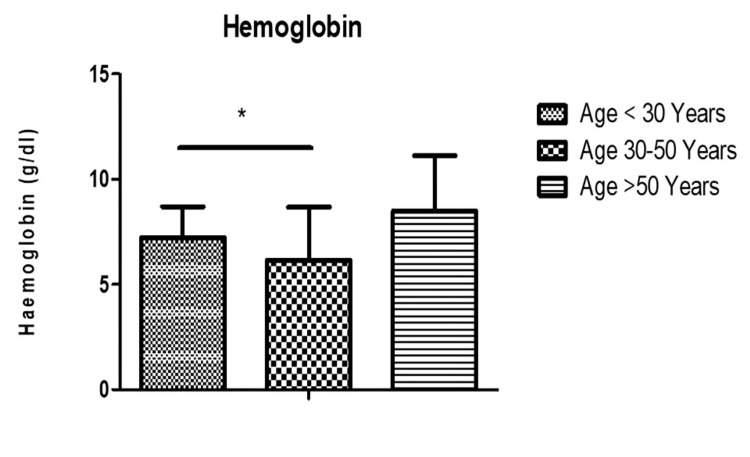
Hb (mean±SD) in the study population. Hb: hemoglobin, SD: standard deviation.

**Figure 3:**
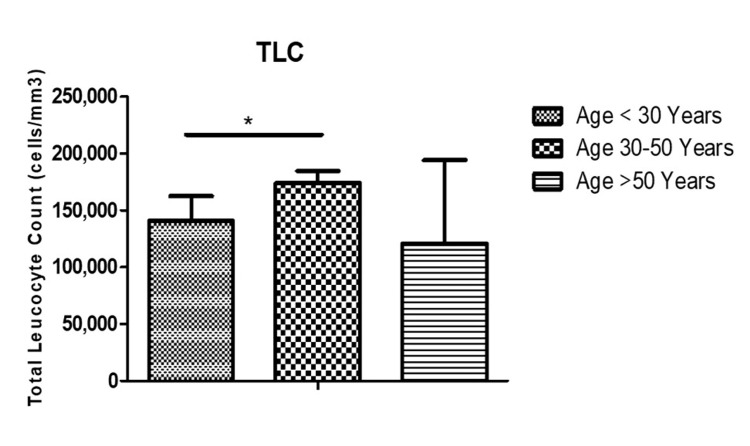
TLC (mean±SD) in the study population. TLC: total leukocyte count, SD: standard deviation.

**Figure 4:**
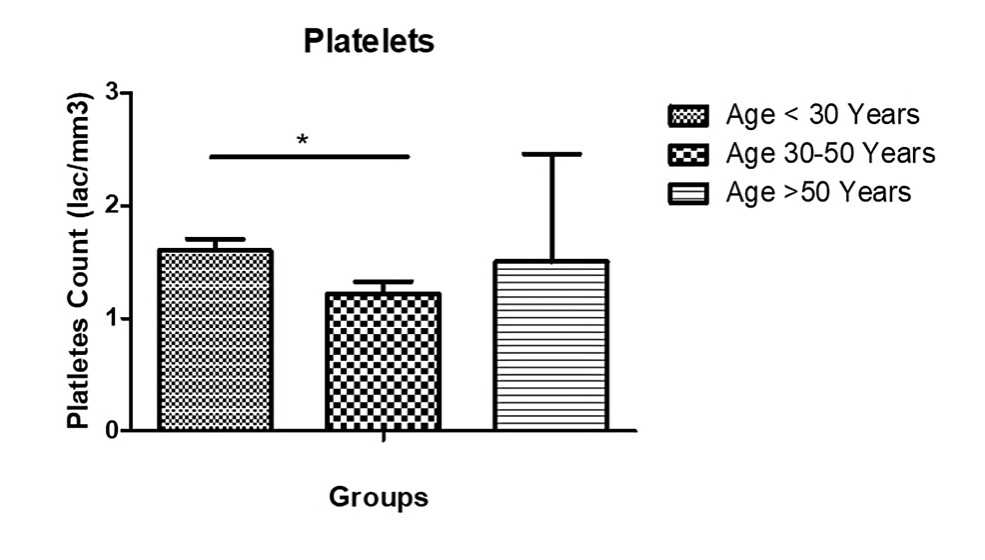
Platelets (mean±SD) in the study population. SD: standard deviation.

The mean±SD of hemoglobin (Hb) in different age groups were as follows: <30 years (7.23±2.4), 30-50 years (6.14±2.5) (P<0.05), and >50 years (8.4±2.6), respectively (Figure 2). Similarly, the mean total leukocyte count (TLC) in the study population was grouped as <30 years (1.4±2.9), 30-50 years (1.7±1.05) (P<0.05), and >50 years (1.2±7.3), respectively (Figure 3). The mean platelet counts in the study population were, respectively, grouped as <30 years (1.6±0.12), 30-50 years (1.2±0.11) (P<0.05), and >50 years (1.5±0.95), respectively (Figure 4).

Chromosomal abnormalities and age groups

Table 2 shows the distribution of patients according to chromosomal abnormalities and age groups, majority of patients between three and 50 years had major abnormalities with n=55 (36.73) at 46, XX, t(9;22) n=15 (27.3), whereas age group <30 years and 30-50 years shows n=46 (31.29) and n=47 (31.97) chromosomal abnormalities.

**Table 2:** Age-based distribution of cytogenetics findings. N: numbers, %: percentage.

Conventional cytogenetic findings	<30 years (N)%	30-50 years (N)%	>50 years (N)%
46,XX	3 (6.5)	14 (25.5)	4 (8.5)
46,XY	14 (30.4)	10 (18.2)	4 (8.5)
46,XY,del11(q23)	3 (6.5)	0	0
46,XY,t(7;15)	2 (4.3)	0	0
46,XY,t(9;22)	0	5 (9.1)	8 (17)
47,XX+18	0	2 (3.6)	0
48,XX,del(9)(p22),+12,+21	3 (6.5)	0	0
52,XY,+1,+8,+10,+17,+21,+21,+22,+mar1	4 (8.7)	0	4 (8.5)
45,XY,del(7)(p15),9,der(9),i(9)(q10),t(9;22)	4 (8.7)	0	4 (8.5)
46,XX,t(9;22)	0	15 (27.3)	0
47,XY,-7,+8,+21,t(9;22)	0	0	4 (8.5)
46,XX,t(1;19)	0	0	4 (8.5)
Not obtained	12 (26.1)	13 (23.63)	15 (31.9)

## Discussion

Acute lymphoblastic (MS1) leukemia is a white blood cell malignancy that is characterized by an excess of lymphoblasts [13]. It is a malignant condition caused by the clonal expansion of immature lymphoid precursors [9]. Acute leukemia is characterized by abnormally fast growth of immature blood cells and incapable to produce healthy blood cells in the BM [14]. The 2008 World Health Organization (WHO) Classification of Hematopoietic and Lymphoid Tissue Tumors includes all specific chromosome abnormalities and their corresponding molecular counterparts [15]. These abnormalities are used alongside morphology, cytochemistry, immunophenotype, and clinical characteristics to define different disease entities within B-lineage ALL (B-ALL). The list contains the numbers 9, 10, and 11 [16]. Although there are several recurring chromosomal abnormalities and gene alterations in T-lineage ALL (T-ALL), they are not yet used to identify separate entities within T-ALL [17].

Demographic and clinical characteristics

The study's findings are believed to have major therapeutic implications, including potential improvements for leukemia treatment. The study offers insights into the genetic evaluation and clinical features of ALL patients, shedding light on demographic variables, clinical manifestations, and cytogenetic abnormalities, and aims to explore the implications of these findings in understanding the disease characteristics and guiding clinical management in the North India population. The study included n=147 ALL patients, with males n=85 (58%) and females n=62 (42%). Demographic variables and clinical features, including complications like fever, pallor, lymphadenopathy, rashes, joint pain, headache, and bleeding manifestations, were assessed. The prevalence of these manifestations provides valuable clinical insights into the presentation and progression of ALL in the studied population [12,18]. However, in a study by Yildirim et al., they reported acute complications in 38 patients, which include infectious complications followed by gastrointestinal complications, drug-related reactions, and thrombotic, neurological, and endocrine/metabolic complications, respectively [19].

Hematological parameters

Hb, TLC, and platelets were analyzed across different age groups. Significant differences were observed in Hb levels among age groups, with lower levels seen in the 30-50 age group compared to <30 years and >50 years. Similarly, TLC and platelet counts exhibited variations across age groups, with the 30-50 age group showing significantly higher TLC compared to other age groups. A study by Patra et al. had a median total leukocyte count (TLC) of 220,000 per cubic millimeter, ranging from 26,810 to 785,430 per cubic millimeter. These hematological parameters reflect the heterogeneous nature of ALL and its impact on hematopoietic function across different age cohorts [12,13]. While it is widely accepted that a decrease in the functioning of hematopoietic stem cells (HSCs) is responsible for age-related hematological issues, there is still a dearth of complete knowledge on the molecular mechanisms underlying these changes [20]. The transcriptional signature observed in studies on aging is associated with alterations in gene expression caused by stress, rather than the process of aging itself [21].

Chromosomal abnormalities

The distribution of patients to chromosomal abnormalities and age groups provides valuable insights into the cytogenetic landscape of ALL. Most patients in the 30-50 age group exhibited major abnormalities, with 46, XX, t(9;22) being the most prevalent abnormality in this cohort. Furthermore, Krishna Chandran et al. identified 388 cases of ALL with a median age of 31 years, of which 16.9% exhibited Philadelphia chromosomes [14,22]. Notably, the prevalence of chromosomal abnormalities was also substantial in the <30 years age group, indicating the diverse genetic heterogeneity across different age ranges [23,24]. Understanding the prevalence and distribution of chromosomal abnormalities can aid in risk stratification and prognostication of ALL patients. Certain abnormalities, such as t (9;22), may have prognostic significance and influence treatment decisions [1,25]. In a study by Li et al., the chromosomal abnormality was identified in 121 out of 3235 couples experiencing recurrent pregnancy loss (RPL), with a prevalence of 3.74%. This included 75 instances in females and 46 cases in males at an individual level. Out of the total number of instances, 101 were identified as structural aberrations. Among them, 46 cases (38.0%) involved balanced translocations, 13 cases (10.7%) involved Robertsonian translocations, and 42 cases (34.7%) involved inversions. Additionally, 20 cases (16.5%) were classified as numerical aberrations [23,26,27].

Limitations

The study's cross-sectional design limits its ability to establish relationships between chromosomal abnormalities and clinical outcomes. The study's sample size of 147 ALL patients may not fully represent the diversity of genetic and clinical characteristics within the North Indian population.

## Conclusions

The genetic evaluation and clinical characterization of ALL patients provide valuable insights into disease presentation, hematological parameters, and chromosomal abnormalities. These findings have important implications for risk stratification, disease monitoring, and treatment planning in ALL. Continued research efforts are essential to unravel the underlying molecular mechanisms driving disease pathogenesis and translate these findings into improved clinical outcomes for ALL patients.

## Appendices

Department Of Clinical Hematology/Anatomy

Cytogenetic Abnormalities in Adults with Acute Lymphoblastic Leukemia (ALL) in North India

Patient registration form

Name ……………………………..………Age/Sex ………Reg. no……………...Date…………

Address…………………………………………………………..Phone no………………………

Clinical Features…………………………………………………………………………………………………………………………………………………………………………………………………………………………………………………………………………………………………………………………………………………………………………………………………………………………………………………………………………………………………………………………………………………………………………………………………………………………

Baseline Investigations

Hb…………..TLC…………………….. DLC……………………………………………………..

Platelets………………………. S Iron/TIBC/Ferritin……………….. S. Bil……………………..

S urea/creat……………….. SGPT/SGOT /SALP…………………S uric acid/LDH……………

GBP…………………………………………………………………………………………………

Special Investigations

BCR-ABL (initial and follow-up)………………………………………………………………….

………………………………………………………………………………………………………

Cytogenetics………………………………………………………………………………………..

Bone marrow………………………………………………………………………………………..

Others……………………………………………………………………………………………….

Diagnosis…………………………………………………………………………………………...
